# A Gelatin Methacrylate-Based Hydrogel as a Potential Bioink for 3D Bioprinting and Neuronal Differentiation

**DOI:** 10.3390/pharmaceutics15020627

**Published:** 2023-02-13

**Authors:** Elisa Marozzi Cruz, Lucas Simões Machado, Laura Nicoleti Zamproni, Larissa Valdemarin Bim, Paula Scanavez Ferreira, Leonardo Alves Pinto, Luiz Antonio Pessan, Eduardo Henrique Backes, Marimélia Aparecida Porcionatto

**Affiliations:** 1 Department of Biochemistry, Escola Paulista de Medicina, Universidade Federal de São Paulo, São Paulo 04039-032, Brazil; 2 Department of Materials Engineering, Universidade Federal de São Carlos, São Carlos 13565-905, Brazil

**Keywords:** bioprinting, GelMA, neurogenesis, neuroprogenitor cells, astrocyte de-differentiation

## Abstract

Neuronal loss is the ultimate pathophysiologic event in central nervous system (CNS) diseases and replacing these neurons is one of the most significant challenges in regenerative medicine. Providing a suitable microenvironment for new neuron engraftment, proliferation, and synapse formation is a primary goal for 3D bioprinting. Among the various biomaterials, gelatin methacrylate (GelMA) stands out due to its Arg-Gly-Asp (RGD) domains, which assure its biocompatibility and degradation under physiological conditions. This work aimed to produce different GelMA-based bioink compositions, verify their mechanical and biological properties, and evaluate their ability to support neurogenesis. We evaluated four different GelMA-based bioink compositions; however, when it came to their biological properties, incorporating extracellular matrix components, such as Geltrex^TM^, was essential to ensure human neuroprogenitor cell viability. Finally, Geltrex^TM^: 8% GelMA (1:1) bioink efficiently maintained human neuroprogenitor cell stemness and supported neuronal differentiation. Interestingly, this bioink composition provides a suitable environment for murine astrocytes to de-differentiate into neural stem cells and give rise to MAP2-positive cells.

## 1. Introduction

Brain injuries are a significant cause of mortality and morbidity worldwide, representing a high cost to healthcare systems [1,2]. Neuronal loss is the ultimate pathophysiologic event in central nervous system (CNS) diseases [3,4], and replacing the lost neurons is one of the major challenges in regenerative medicine. In this scenario, adult neurogenesis is a critical process in CNS repair. Since endogenous adult neurogenesis is limited [5], many attempts to stimulate this process or to deliver exogenous neural stem cells are relevant strategies [6]. Cell engraftment, function, and behavior are governed by the tissue’s native microenvironment and rely on mechanical and biochemical features, such as topology, stiffness, and chemical cues [7,8]. However, the lesion microenvironment is hostile to the repair process [9].

Therefore, proper modeling and producing artificial structures that mimic the CNS microenvironment is crucial for better understanding the mechanisms involved in the healthy and injured brain [10,11]. The bioprinting technology appears to be an innovative technique to produce 3D tissue-like structures as an alternative to traditional 2D models and is a potentially powerful tool for repairing CNS injuries [11]. Bioprinted constructs have tunable features, such as incorporating different cell types at varied densities, different construct geometries, and cell–matrix interfaces based on personalized bioink composition [12]. Although 3D bioprinting studies are rapidly growing, the application of 3D bioprinting in neural tissue engineering is still very limited [13,14]. The challenges in printing neural tissues are associated with high criteria for developing a bioink that meets both the physical requirements and a suitable biological environment [15]. A conflict exists in constructing a soft environment, which is essential for the survival and proliferation of neural cells or neural progenitors, that can keep high shape fidelity after its extrusion [16]. In addition, some significant challenges remain to be addressed, such as the efficiency of the printing process, reduced cell viability, and minimal cell–material interaction [17].

Gelatin methacrylate, or GelMA, a chemically modified gelatin obtained by adding methacrylic anhydride, is widely used to produce hydrogels due to its photocurable feature in the presence of a photoinitiator. Moreover, GelMA stands out due to the presence of Arg-Gly-Asp (RGD) domains and target sites for matrix metalloproteinase, which assure its biocompatibility, cell remodeling and degradation under physiological conditions, as well as its fine-tunable mechanical features [18,19,20]. Mechanical properties are of significant interest since they can play different roles during cell proliferation, migration, and differentiation due to the mechanotransduction effect [21,22,23]. This work aimed to produce different GelMA-based bioink compositions, verify their mechanical and biological properties, and evaluate their ability to support neurogenesis.

## 2. Materials and Methods

### 2.1. Reagents

Cell Culture: Geltrex™ (#A1413302, Gibco, Grand Island, NY, USA), Irgacure (#2959, Sigma-Aldrich, St. Louis, MI, USA), Trypsin (#15090-046, Sigma-Aldrich), Fetal Bovine Serum (#12657-029, Gibco), glutamine (#101806, MP Biomedicals, Santa Ana, CA, USA), penicillin/streptomycin (#15140122, Gibco), supplemented Dulbecco’s Modified Eagle Medium (DMEM)/F12 (#12500-062, Gibco), Essential E8 medium (#A15169-01, Gibco), Neurobasal medium (#21103-049, Gibco), B27 without vitamin A (#12587-010, Gibco), B27 (#1750-044, Gibco), N2 (#17502-048, Gibco), Glutamax (#3505-061, Gibco), SMAD LDN193189 (# SML0559, Sigma-Aldrich), SB431542 (#S4317, Sigma-Aldrich), StemPro Accutase (#A11105-01, Gibco), epidermal growth factor (EGF, E9644-2MG, Sigma Aldrich), basic fibroblast growth factor (bFGF, #PHG0026, Gibco), poly-L-ornithine (#P4957, Sigma-Aldrich), laminin (#L2020, Sigma-Aldrich), ascorbic acid (#A4403, Sigma-Aldrich), db-cAMP (#D0627, Sigma-Aldrich), Brain Derived Neural Factor (BDNF, #B3775, Sigma-Aldrich), Glial Derived Neural Factor (GDNF, #G1777, Sigma-Aldrich), normal goat serum (#S26-100 mL, Millipore, Burlington, MA, USA),T25 culture flasks (#430168, Corning, Corning, NY, USA), T75 culture flasks (#430720U, Corning), 100 mm^2^ culture plates (#93100, TPP, Trasadingen, Switzerland). Viability assay: Resazurin (#R7017, Sigma-Aldrich).

PCR: TRIzol^TM^ (#15596026, Thermo Fischer, Waltham, MA, USA), SuperScript™ III First-Strand Synthesis SuperMix (#18080400, Thermo Fischer), Fast SYBR™ Green Master Mix (#4385612, Thermo Fischer).

Antibodies: DCX (#ab18723, Abcam, Cambridge, UK), TUBB3 (#MA1-118, Thermo Fischer), SOX2, (#ab79351, Abcam), MAP2 (#PA5-17646, Thermo Fischer and #ab5622, Millipore, USA) or GFAP (#ab5541, Millipore), DAPI (#62248, 1:500, Sigma-Aldrich), AlexaFluor 488 (#A21441, Invitrogen, Waltham, MA, USA), AlexaFluor 594 (#A21203, Invitrogen), AlexaFluor 647 (#A21449, Invitrogen).

### 2.2. Bioink

In this work, different GelMA-based bioink compositions were produced with the aim of assessing their potential to support neurogenesis. We tuned their mechanical and biological properties by modifying the proportions of the crosslinkable phase (GelMA) and gelatin. GelMA was synthesized according to the recently published protocol [24]. Prior to use, lyophilized gelatin and GelMA were diluted in PBS at different proportions. The photoinitiator Irgacure was added to the bioink at a concentration of 0.5 wt.%, and the prepared bioink was incubated in an oven a 37 °C in the dark for at least 4 h before use. The bioinks were sterilized using a polypropylene 0.22 μm filter for biological tests. The addition of 50 v% of Geltrex™ was also investigated with the aim to increase cell viability. GelMA crosslinking was carried out by exposing the construct to ultraviolet (UV) light (20 mm distant from UV source) at 2 mW cm^−2^ for 5 min.

The tested bioink compositions are displayed in Table 1.

### 2.3. Characterization of the Mechanical Properties of Scaffolds

The mechanical properties of the hydrogels were determined through compression tests carried out in an Instron (Model 6659, High Wycombe, UK), with a load cell of 5 N, preload of 10 mN, strain rate of 1.3 mm s^−1^, and temperature of 23 °C. The compression modulus was calculated from linear regression in 0.05–0.25% and 10–20% regions. The samples were manufactured by casting the bioink in 35 mm Petri dishes (approximately 2.5 mL per dish) and photocuring them in a UV system for 5 min before analysis. Compression tests were performed on stamped samples (7 mm diameter), and their dimensions were measured before each analysis: height (2–2.2 mm) and diameter (~7 mm). Mechanical analysis was performed on at least five samples for each hydrogel sample. A representation of the materials and apparatus used to manufacture the hydrogels is presented in Supplementary Figure S1.

### 2.4. Degradation Test

The degradation test aims to determine the stability of the material after its physical and chemical crosslinking process. The degradation test consists of weighing the samples after the crosslinking process; then, the samples were immersed in PBS, 10 mM, pH 7.4 at 37 °C, for different periods of times, namely 1, 3 and 7 days. The samples were recovered with a spatula, gently dried on paper and weighed on a scale with a resolution of 0.001 g. The mass loss for the different analysis times was calculated from Equation (1). Three samples were analyzed for each period.

(1) $$∆m\;\left(\%\right)=\frac{m_{i}-m_{f}\;}{m_{i}}*100$$

where ∆m is the weight loss percentage, $m_{i}$ stands for initial mass and $m_{f}$ for the mass at each time point.

### 2.5. Rheological Analysis

Rheological analyses were assessed in a stress-controlled rheometer (AR1500ex, TA Instruments, New Castle, DE, USA) under the following conditions: parallel plate (50 mm sand-blasted), 500 mm gap. The tests were conducted under permanent and oscillatory regimens (temperature sweep). The permanent regime, assessed to determine the flow properties of the bioink, was conducted at 25 °C from 0.1 to 100 s^−1^. The points were collected in triplicates, with a 5% maximum deviation among them or a maximum assay time per point of 15 s. A logarithmic ramp was used. For oscillatory analysis, storage (G′) and loss (G″) moduli were obtained as a function of the temperature ranging from 37 to 10 °C, with a cooling rate of 2 °C min-1 and at linear viscoelastic region (LVR, oscillating strain of 1% and frequency of 1 Hz).

### 2.6. Microstructural Analysis

Scanning electron microscopy (SEM) was used to investigate the morphology of cryogenic fractures of the lyophilized bioink. The analyses were performed using a Philips SEM (FEI company, Hillsboro, OR, USA), model XL30 FEG, operating at a voltage of 2 kV. The cryofractured samples were adhered to aluminum stubs and covered with a thin layer of gold to enable the analysis.

### 2.7. Biological Characterization

A summary and flow of all performed biological tests is presented in Supplementary Figure S2.

### 2.8. Extraction and Cultivation of Murine Cortical Astrocytes

One-day-old C57bl/6 mice were obtained from the Institution’s animal facility (CEDEME—Ethics Committee in the Use of Animals approval CEUA 2432170220). The extraction protocol was described by Schildge et al. [25]. Briefly, after dissection, the cortical tissue was mechanically digested in a HBSS solution with micro-scissors and decanted, then enzymatically digested using 1X Trypsin. After trypsin neutralization with an equal volume of Fetal Bovine Serum (FBS), the cell suspension was centrifuged at 300 g for 5 min. The resulting pellet was resuspended in 1 mL of Dulbecco’s Modified Eagle Medium (DMEM)/F12 supplemented with 10% FBS, 2% glutamine and 1% penicillin/streptomycin (PS) and transferred to a T25 culture flask. Medium volume was adjusted to 4 mL, and cells were maintained in culture until the second or third passages in T75 culture flasks or 100 mm^2^ culture plates.

To induce astrocyte reactivation, a 200 µL pipette tip was used to scratch the surface of the culture plates, mimicking a mechanical lesion [3,26,27]. Astrocytes were kept in culture for three days after reactivation induction and then used for further experiments.

### 2.9. Human Induced Pluripotent Stem Cell (hiPSC) Culture and Human Neural Progenitor Cell (hNPC) Differentiation

The human induced pluripotent stem cells (hiPSCs) used in this work were generated and graciously donated by the National Embryonic Stem Cell Laboratory (LaNCE), coordinated by Dr. Lygia V. Pereira. The hiPSCs were generated from human erythroblasts reprogrammed by episomal vectors containing the Yamanaka factors Sox2, Oct 3/4, cMyc, and Klf-4. The hiPSCs were cultured in 6-well Geltrex™-coated plates in Essential E8 medium. The medium was changed daily until cells were subjected to the differentiation protocol.

The human neural progenitor cell (hNPC) lineage was derived from hiPSCs by dual SMAD inhibition [28,29,30,31]. At passage 17 (P17), cells were detached using 0.5 mM EDTA and seeded onto a Geltrex^TM^-coated well to reach 100% confluency the next day. The following day, E8 medium was replaced by neural induction medium (NIM) that consisted of 50% Neurobasal medium and 50% DMEM/F12 supplemented with B27 without vitamin A, N2, 1 mM Glutamax, 1% PS, SMAD LDN193189 (0.1 μM,) and SB431542 (10 μM,). Medium was changed daily until day 14, when cells were passaged with StemPro Accutase onto 6-well Geltrex^TM^-covered plates.

After the induction, the hNPCs were cultured in neural expansion media (NEM), composed of 50% Neurobasal medium and 50% DMEM/F12 supplemented with B27 without vitamin A, N2, 1 mM Glutamax, 1% PS, 20 ng mL^−1^ of epidermal growth factor (EGF) and 20 ng mL^−1^ basic fibroblast growth factor (bFGF). NEM was changed every other day and cells were passaged with StemPro Accutase when needed. After 3 passages, the Geltrex^TM^ coating was replaced by poly-L-ornithine and laminin-coated wells.

A successful induction was confirmed by immunofluorescence analysis of known neural progenitor markers such as NESTIN, and RT-qPCR expression analysis indicated by decreased expression of pluripotency-associated genes such as *OCT4* and *NANOG* and increased expression of NPC-associated genes such as *NESTIN*, *SOX2*, *PAX6*, *FOX1G*, and *TBR2* (Supplementary Figure S3).

### 2.10. Neuroblastoma Cells (SH-SY5Y)

The human neuroblastoma SH-SY5Y (#CRL-2266, ATCC, Manassas, VA, USA) cell line was cultured in high-glucose DMEM, supplemented with 10% FBS, 2% glutamine, and 1% PS.

### 2.11. hNPC and SH-SY5Y Viability in Bioink

Briefly, hNPCs and neuroblastoma cells (SH-SY5Y) were resuspended in the bioinks with different compositions at densities of 5 × 10^6^ cells mL^−1^ (hNPCs) and 2 × 10^6^ cells mL^−1^ (SHSY5Y). For viability tests, cells were plated in 20 µL of culture medium (2D) or 20 µL of bioink. After UV-induced reticulation, cells were allowed to attach overnight. Then, samples were transferred to a new culture plate and medium was changed every 2–3 days during the analysis period (6 days). Resazurin assay was used to assess cell proliferation according to the manufacturer’s protocol. On the second day of culture, the previous medium was removed at each time point and substituted by 500 µL of a 10% Resazurin solution diluted in culture medium. Samples were incubated in the dark for 24 h. Three controls containing cell-free Resazurin solution were also incubated (negative control). Subsequently, 100 µL of the solution of each sample was transferred to a new plate for absorbance endpoint measurement using the plate reader SpectraMax^®^ M3 (570 nm) (Molecular Devices, San Jose, CA, USA). The assay was repeated on the 5th and 6th days (Supplementary Figure S2A). After that time, RNA extraction from the cells in the bioink was conducted. The percentage of live cells was calculated based on the 2D control (considered as 100% of cell viability).

### 2.12. hNPC and SH-SY5Y Gene Expression in Bioink

After 6 days of culture, RNA was extracted using TRIzol^TM^ reagent (Supplementary Figure S2A), and cDNA was produced with the SuperScript™ III First-Strand Synthesis SuperMix, according to the manufacturer’s protocol. The primers used are shown in Table 2. qPCR was conducted using Fast SYBR™ Green Master Mix. Relative expression calculation was performed by the 2^−ΔΔCt^ method [32], using the geometric mean of two endogenous genes (Beta-actin and GAPDH) as reference controls.

### 2.13. 3D Bioprinting of Murine Cortical Astrocytes and hNPCs

The protocol for 3D bioprinting was adapted from de Melo et al. [23]. The bioink 4GMA_Gx was used for all experiments in this section. Both reactive and non-reactive astrocytes were resuspended in the bioink at a density of 4 × 10^6^ cells mL^−1^, while the hNPCs at P7 were resuspended at a density of 14.5 × 10^6^ cells mL^−1^. Cells were bioprinted using a 3DBS Educational Starter Printer, which produced constructs measuring 4 × 4 × 1 mm. For bioink crosslinking, all constructs were exposed to UV light for 5 min and transferred to 24-well culture plates for cultivation.

Bioprinted hNPCs were maintained in neural maturation media (NMM), described in further detail in the next section (Supplementary Figure S2B). Both 3D-bioprinted reactive and non-reactive astrocytes were divided and maintained in two different media: astrocyte cell medium (AST; DMEM F12 supplemented with 10% FBS, 2% glutamine, and 1% PS) or neural stem cell medium (NSC; DMEM F12 supplemented with 2% B27, 1% glutamine, 1% PS, 10 µg mL^−1^ EGF, 10 µg mL^−1^ bFGF, and 20 µg mL^−1^ heparin) (Supplementary Figure S2C).

Murine cells in the constructs were analyzed by immunofluorescence at 3, 5, and 10 days post-printing (dpp) or 28 dpp for hNPCs.

### 2.14. Neuronal Induction and Maturation

The neurogenic potential of non-reactive astrocytes was tested by inducing neural differentiation at 10 dpp (Supplementary Figure S2D). To achieve this, the NSC medium was replaced by medium without growth factors (EGF and bFGF), supplemented with 10 µM retinoic acid. Constructs were prepared for immunofluorescence analysis 7 and 14 days after differentiation induction.

The hNPC maturation was based on previously published work [27,29,30]. The generated constructs were maintained in neural maturation media (NMM), composed of 50% Neurobasal and 50% DMEM/F12, supplemented with B27 without vitamin A, N2, 2 mM Glutamax, 1% PS, ascorbic acid (80 µM), db-cAMP (50 µM), 20 ng mL^−1^ Brain Derived Neural Factor (BDNF) and 10 ng mL^−1^ Glial Derived Neural Factor (GDNF). The media was changed every three days.

### 2.15. Immunofluorescence Analysis of Cell Markers

For immunofluorescence analysis of specific markers in bioprinted cells, cell medium was removed, and samples were washed with PBS, 10 mM, pH = 7.4, for 5 min and then fixed with 4% paraformaldehyde for 40 min. The constructs were washed 3 times with PBS for 5 min each and incubated with 0.1 M L^−1^ glycine solution for 15 min. After 3 washes with PBS of 5 min each, samples were blocked with 5% (*v*/*v*) normal goat serum in PBS containing 0.1% Triton X-100 for 1 h. The constructs were then incubated with the following primary antibodies overnight: DCX, TUBB3, SOX2, MAP2 or GFAP. Samples were then washed with PBS three times and then incubated with DAPI and the following secondary antibodies (1:500) for 2 h: AlexaFluor 488, AlexaFluor 594, or AlexaFluor 647. The samples were analyzed using confocal microscopy (Leica, TCS SP8 CARS, Wetzlar, Germany).

### 2.16. Statistical Analysis

Data were analyzed using GraphPad Prism 9.0 (GraphPad software, San Diego, CA, USA) and presented as the means ± SD. The treatment groups were evaluated by one-way analysis of variance (ANOVA) and, if mentioned, with the Tukey post hoc test. In all tests, only *p* < 0.05 was considered to indicate a significant difference between groups.

## 3. Results

### 3.1. Mechanical Properties of the Bioink

**Figure 1:**
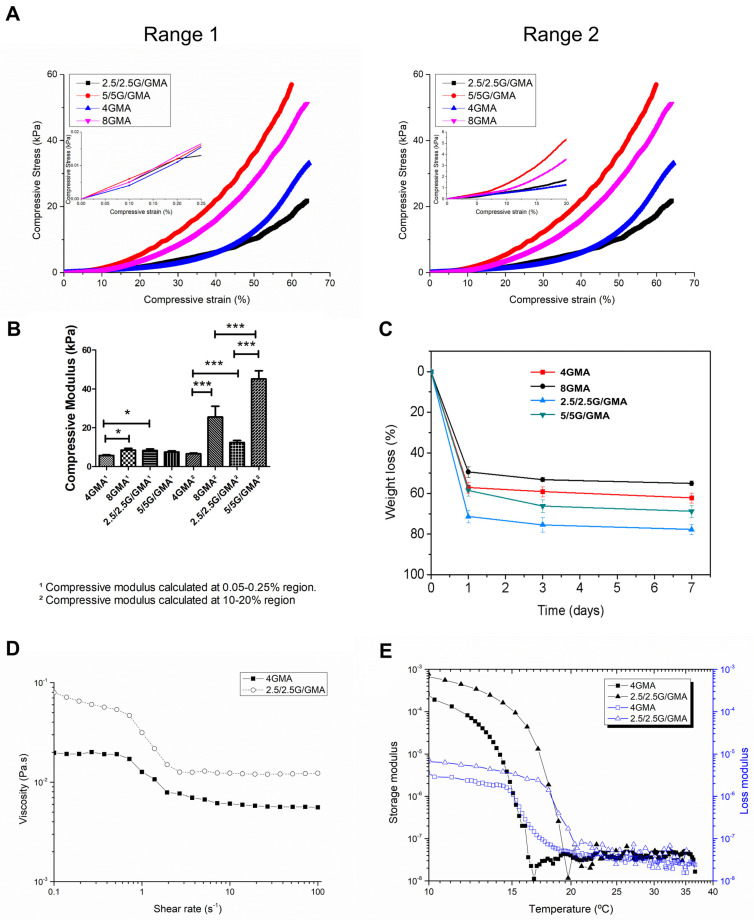
Mechanical and rheological properties of the bioink. Mechanical properties of the bioink under compressive loading (Range 1: zoom of the 0.05–0.25% region, Range 2: zoom of the 10–20% region) (**A**). Compressive moduli of the bioink under compressive loading (**B**). Bioink degradation (**C**). Viscosity versus shear rate for the bioink analyzed at 25 °C (**D**). Storage and Loss moduli versus temperature for the bioink analyzed (**E**). * = *p* < 0.05, *** = *p* < 0.005, one-way Anova plus Tukey post hoc test.

Mechanical properties are of major interest since they can alter cell fate due to mechanotransduction effects [22]. The curves of compressive stress versus compressive strain for the bioinks we produced are represented in Figure 1A. None of the samples fractured even at high deformation, i.e., higher than 50% of compressive strain. Although the bioinks seem to present similar behavior at low deformation (Figure 1A, range 1, maximized area), their mechanical properties present significant variation after 10% deformation (Figure 1A, range 2, maximized area); therefore, the compressive moduli were calculated at two different ranges using linear regression: 0.05–0.25% and 10–20% (Table 3). It is worth noting that, despite using a low-profile cell load and preload of 10 mN, the materials presented significant dispersion values. The compressive moduli calculated at first range (0.05–0.25%) almost did not change since the low deformation is not capable of evidencing the differences between the bioinks, except for the comparisons between 4GMA and 8GMA (*p* < 0.05), and 4GMA and 2.5/2.5G/GMA (*p* < 0.05) (Figure 1B). The mechanical characterization of the bioink at the second range (10–20%) shows that, in these compositions, the addition of gelatin has a higher impact on the mechanical properties. The 2.5/2.5G/GMA compressive modulus increases by almost 50% when calculated in a different range, and a more expressive tendency is observed by 5/5G/GMA. In contrast, the 4GMA presented a practically linear behavior, and there is only a slight difference between the compressive modulus at the two ranges.

### 3.2. Degradation Test

This test aimed to simulate the biodegradation behavior of the various bioinks under physiological conditions and their potential as candidates for bioprinting. Some properties that impact the biodegradation kinetics are related to the hydrogel crosslinking degree and concentration of the cross-linkable phase. On the first day, all the bioinks underwent severe weight loss. That can be explained by the loss of the non-crosslinked phase (gelatin). Accordingly, the compositions with higher cross-linkable phase (GelMA) presented higher residual mass at 7 days (2.5/2.5G/GMA < 5.0/5.0G/GMA < 4GMA < 8GMA) (Figure 1C). Furthermore, a substantial modification of the biological and mechanical properties occur as the biodegradation proceeds since the soluble phase gelatin either in GelMA or not, is leached out.

### 3.3. Rheology Behavior

The measurement of bioink viscosity versus shear rate and oscillatory temperature sweep is represented in Figure 1D,E, respectively. Regarding the flow properties, both bioinks are very fluid with a low viscosity level and present a pseudoplastic behavior, whereas at low shear rates, the 4GMA bioink showed a Newtonian plateau and a power law behavior around 1 s^−1^. On the other hand, 2.5/2.5G/GMA presented a decrease in viscosity from a low shear rate (0.01 s^−1^) which intensified at a shear rate of 1 s^−1^. In both cases, higher rates resulted in a second Newtonian Plateau, and the viscosity remained practically unchanged with the shear rate.

The viscoelastic characterization of the bioinks was performed by determining their elasticity (storage modulus—G′) and viscosity (loss modulus—G″) under different cooling; this analysis allows the determination of the gelling point where G′ > G″, i.e., the bioink behaves more solid-like and is capable of retaining its form precisely. Both 2.5/2.5G/GMA and 4GMA bioinks present different gelation temperatures around 17 °C and 15 °C, respectively. This result shows that adding gelatin to the composition leads not only to gelation at higher temperatures but also produces a higher G′. This behavior has already been observed during mechanical characterization, where this composition showed a higher compressive modulus than 4GMA.

### 3.4. Bioink Microstructural Analysis by SEM

**Figure 2:**
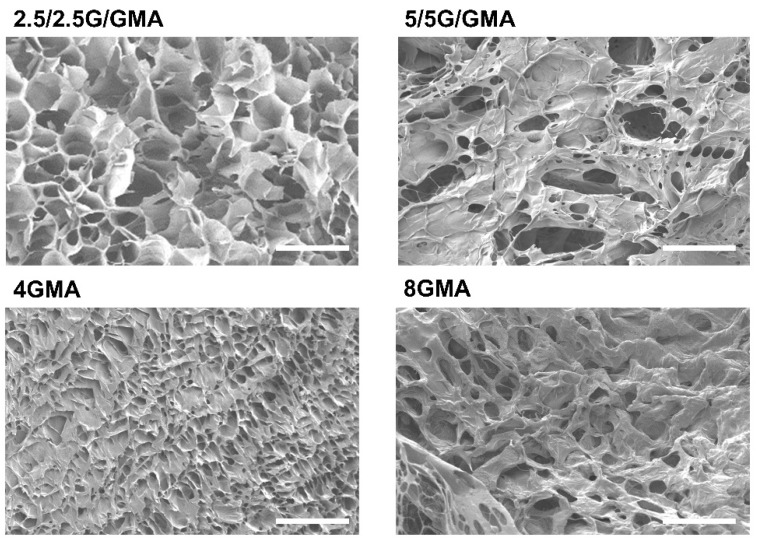
Micrographs of lyophilized bioinks obtained by SEM analysis (Magnification of 500× and 50 µm scale).

The qualitative analysis of the micrographs shows that the more rigid bioinks, such as 5/5G/GMA and 8GMA, tend to present a less porous morphology, while 2.5/2.5G/GMA and 4GMA exhibited higher porosity (Figure 2). This behavior is associated with the contents of a cross-linkable phase, GelMA, that leads to lower porosity. It is worth mentioning that the samples were analyzed after the crosslinking process and thereafter immersed in liquid nitrogen to freeze the microstructure. However, during the lyophilization process, slight modifications in the construct morphology can occur during water removal.

### 3.5. The Addition of Geltrex^TM^ to the Bioink Is Essential for Maintaining hNPC Viability

**Figure 3:**
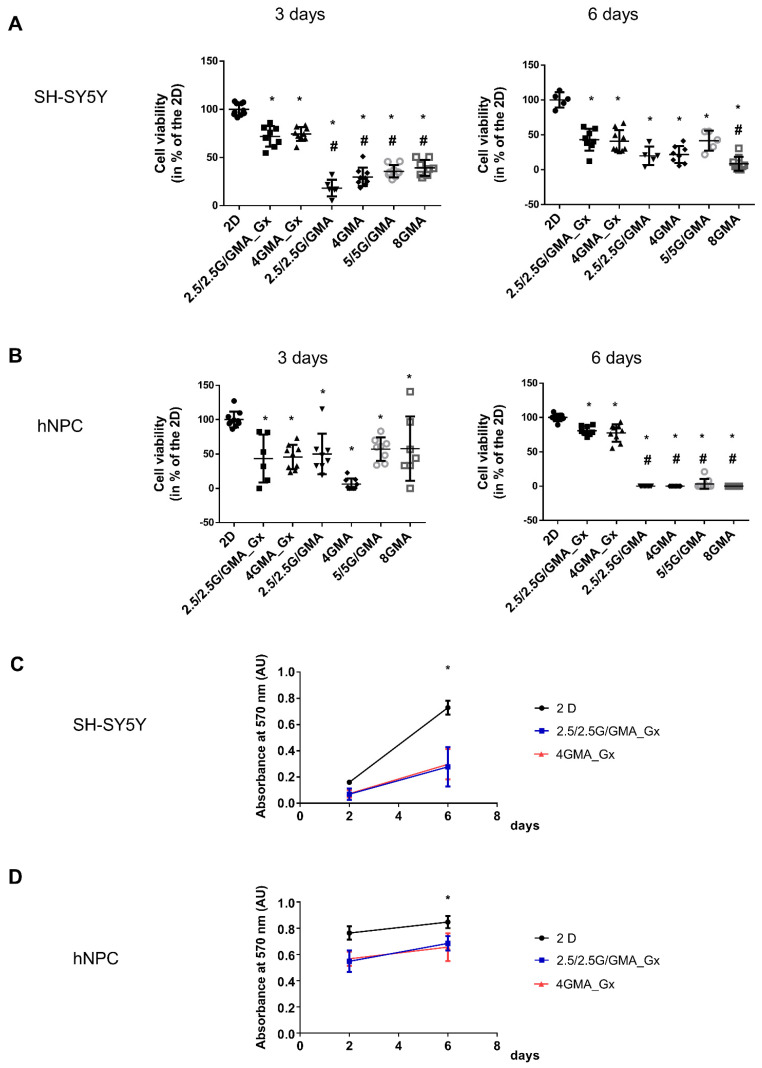
The addition of Geltrex^TM^ to the bioink is essential for maintaining hNPCs. The viability SH-SY5Y in all bioinks tested was below 2D viability. On the sixth day, viability in all bioinks remained below 2D viability. Geltrex^TM^ addition to the bioink slightly increased the mean viability, but it was only statistically higher in comparison to the 8GMA group (**A**). For hNPCs, on the third day, there was a significant decrease in cell viability in all groups compared to the 2D and the Geltrex^TM^-added groups. On day six, only bioinks containing Geltrex^TM^ added to their composition were able to maintain hNPC viability, although at lower levels than for 2D (**B**). Comparison of absolute absorbances on day three and 6 shows an increase in the number of viable cells, suggesting cell proliferation for both SH-SY5Y (**C**) and hNPCs (**D**). * = *p* < 0.05 in comparison to 2D group, # = *p* < 0.05 in comparison to both 2.5/2.5G/GMA_Gx and 4GMA_Gx, one-way Anova plus Tukey post hoc test.

The next step was verifying whether the bioink was suitable for sustaining neural cell growth. First, the viability of human neuroblastoma cells (SH-SY5Y) on the different bioinks was tested. On the third day, the cell viability for all bioinks tested was below 2D viability; however, no significant differences among the groups were observed. On the sixth day, the viability for all bioink tested remained below 2D viability; nonetheless, adding Geltrex^TM^ to the bioink slightly increased the mean cell viability, but it was only statistically higher compared with the 8GMA group (Figure 3A). However, moving to hNPCs, it is possible to verify that, already on the third day, there was a significant decrease in cell viability in all groups compared to Geltrex^TM^-added groups. The result was more evident on day six when only the Geltrex^TM^-added bioink could maintain hNPC viability (Figure 3B). In accordance with that result, it is possible to verify that the hNPC morphology in the Geltrex^TM^-added bioink is much more similar to the morphology observed in 2D cultures, with the presence of cell extensions in comparison with the round shape observed in bioink without Geltrex^TM^ (Supplementary Figure S4).

When comparing the absolute value of absorbance for both SH-SY5Y and hNPCs at three and six days, it is possible to observe that, in all three conditions, 2D, 2.5/2.5G/GMA_Gx and 4GMA_Gx, there was an increase in the absolute absorbance value, suggesting that cells were able to proliferate in the Geltrex^TM^-added bioink (Figure 3C,D).

### 3.6. Cells Cultured in Bioink for Six Days Present Minor Changes in Their Gene Expression Profile

**Figure 4:**
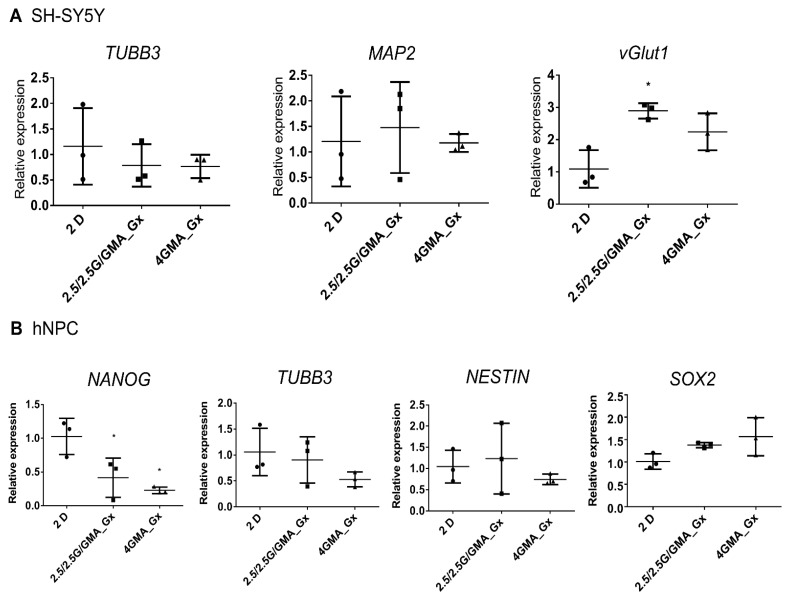
Cells cultured in bioink during 6 days present minor changes in their gene expression profile. In SH-SY5Y, the expression of the vesicular glutamate transporter 1 (vGlut1) was slightly increased in s2.5/2.5G/GMA_Gx (**A**). For hNPCs, there was a significant reduction in NANOG expression in both 2.5/2.5G/GMA_Gx and 4GMA_Gx compared to the 2D control (**B**). * = *p* < 0.05, one-way Anova plus Tukey post hoc test.

Neuronal and stem cell markers were assessed to check whether bioinks stimulate changes in cell fate. In SH-SY5Y, the expression of the vesicular glutamate transporter 1 (vGlut1) was slightly increased in the 2.5/2.5G/GMA_Gx (Figure 4A), which could represent an increase in glutamatergic activity.

For hNPCs, there was a statistically significant reduction in NANOG expression in both 2.5/2.5G/GMA_Gx and 4GMA_Gx compared to the 2D control. However, no statistically significant modification of the other pluripotency markers (SOX2 and Nestin) was seen, nor was there an increase of neuronal markers (TUBB3) (Figure 4B), indicating that the hNPCs were able to keep their stemness potential in the bioinks.

### 3.7. Bioprinted hNPCs Successfully Differentiated in Immature Neurons

**Figure 5:**
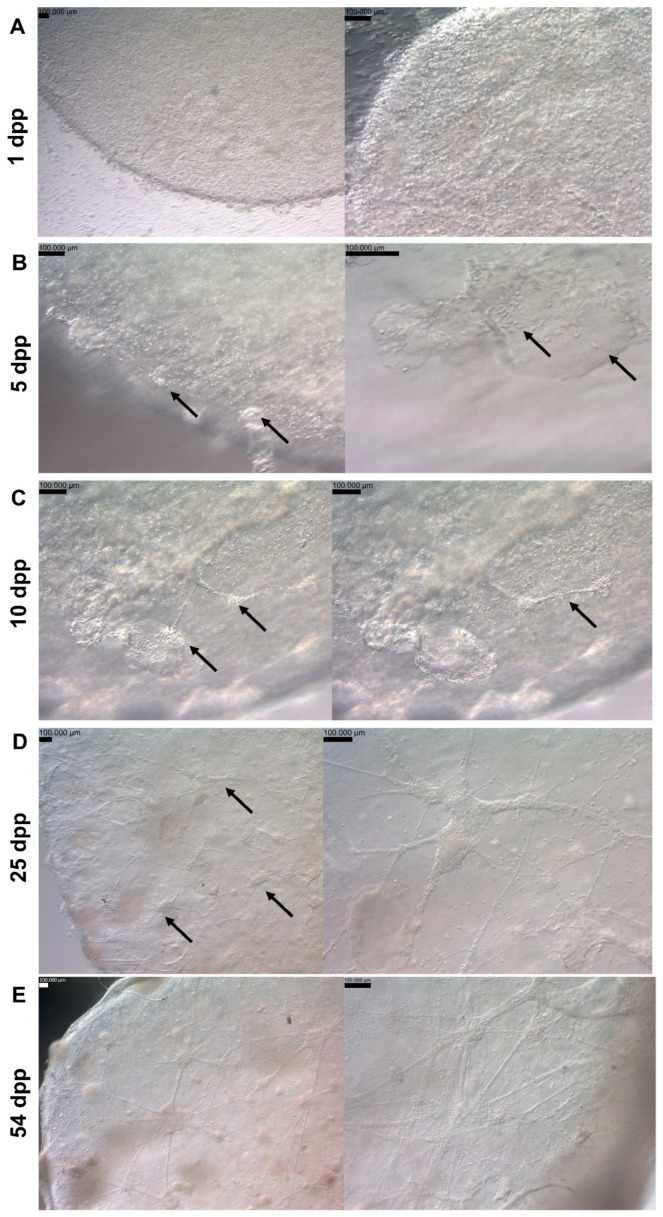
Bioprinted hNPCs’ change of morphology over time. Light microscope images of 3D-bioprinted hNPCs in neural maturation media 1 dpp (**A**). Light microscope image of 3D-bioprinted hNPCs in neural maturation media 5 dpp. Cells present a more NPC-like morphology (arrows) and it is possible to observe the formation of cell clusters in the constructs (arrows) (**B**). Light microscope images of 3D-bioprinted hNPCs in neural maturation media 10 dpp at the same position but at different focus lengths. Cell clusters (arrows) have increased in size, occupied different layers in the constructs and connected through cell protusions (**C**). Light microscope images of 3D-bioprinted hNPCs in neural maturation media 25 dpp. Connected cell clusters throughout the whole construct are observed (arrows). Aside the cluster, it is possible to see cells with different morphology among the construct, with long projections and apparent connections (**D**). Light microscope images of 3D-bioprinted hNPCs in neural maturation media 54 dpp showing connected cell clusters throughout the whole construct (**E**). Scale bar = 100 µm. dpp = days post-printing.

Based on each bioink performance, we selected the 4GMA_Gx composition for further bioprinting assays. First, hNPCs were bioprinted and maintained in neural maturation media (NMM). Most cells had a rounded morphology on the 1st day post-printing (dpp) (Figure 5A). However, it was possible to observe a small number of cells throughout the construct with a more elongated shape, similar to what is expected in a 2D culture (Supplementary Figure S5A). Over the next few days, more cells changed from rounded to elongated, along with even longer protrusions. At the same time, small cell clusters became noticeable at the edges of the constructs (Figure 5B).

At 10 dpp, there was an apparent increase in the number of clusters that became larger, occupying different layers of the constructs and connecting with one another via elongations from the cells inside the clusters (Figure 5C). It was also possible to see cells outside the clusters with even longer processes and characteristics associated with NPC morphology (Supplementary Figure S5B).

**Figure 6:**
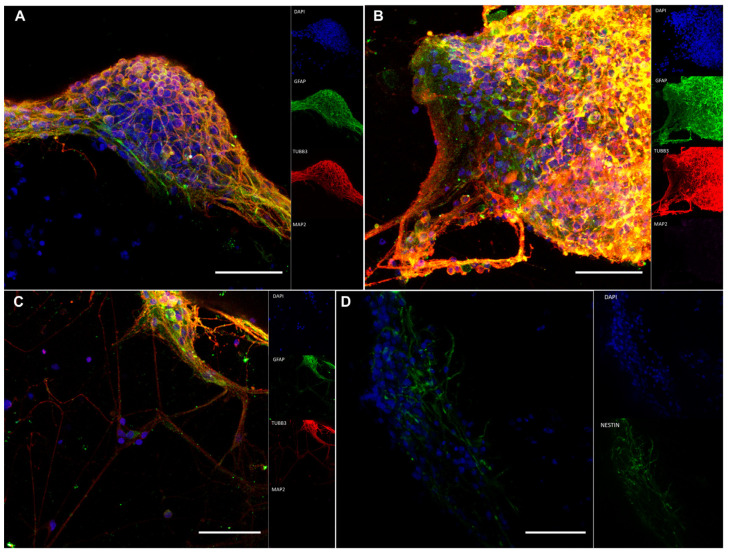
Bioprinted hNPCs successfully differentiated in immature neurons. Immunofluorescence for GFAP, TUBB3 and MAP2 markers in bioprinted hNPCs on neural maturation media at 28 dpp (**A**,**B**). Cells outside the clusters that are marked strongly for TUBB3 rather than GFAP (**C**). Immunofluorescence for NESTIN in bioprinted hNPCs on neural maturation media at 28 dpp (**D**). Scale bar = 50 µm. dpp = days post-printing.

At 25 dpp, the clusters were observed throughout the whole extension of the constructs (Figure 5D). Interestingly, cells with slightly different morphologies were observed outside the clusters (Figure 5D). Immunofluorescence on 25 dpp constructs showed many cells positive for both GFAP, a known neural progenitor marker, and TUBB3, a marker of mature neurons. However, there were also cells positively marked for either one or the other (Figure 6A–C). Interestingly, NESTIN (also an NPC marker) was not as strongly present as GFAP (Figure 6D). All constructs were negative for MAP2, a mature neuron marker.

The constructs have been successfully cultured over 54 dpp (Figure 5E). The cells continued to form elongations linking neuron-like cells in the construct (Supplementary Figure S5C). In some cases, it was possible to see the formed clusters in the constructs without the aid of a microscope (Supplementary Figure S5D).

### 3.8. Bioprinted Murine Astrocytes De-Differentiated in NSC and Originate New Neurons

Both non-reactive and reactive murine astrocytes were bioprinted and their behavior was observed in two different media (AST or NSC). When cultured in AST media, non-reactive bioprinted astrocytes presented a round morphology in contrast with the reactive astrocytes’ star shape observed in 2D. SOX2 expression was present in reactive astrocytes at 5 dpp, which was not observed in the subsequent timepoint or in the non-reactive astrocyte group. No groups showed expression of DCX (doublecortin) (Supplementary Figure S6).

**Figure 7:**
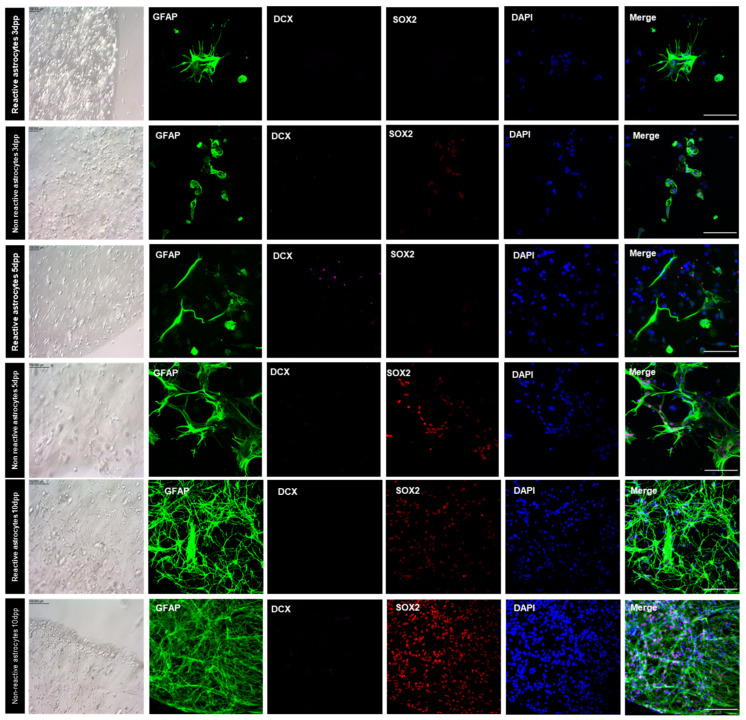
Immunofluorescence analysis of GFAP-, SOX2-, DCX-positive cells in bioprinted murine reactive and non-reactive astrocytes under influence of NSC culture media. While SOX2-positive nuclei were only observed in 3D-bioprinted reactive astrocytes at 10 dpp, this was noted at all timepoints for non-reactive astrocytes. In this group, cells formed a net throughout all constructs, as the majority of DAPI-stained nuclei were SOX2 positive. Scale bar (bright-field) = 100 µm. Scale bar (fluorescence) = 50 µm. dpp = days post-printing.

However, when bioprinted astrocytes were cultivated in NSC media, they developed a more elongated morphology from 1 dpp, as shown in Figure 7. Throughout all 10 days post-printing, cells spread throughout the bioprinted construct and at the edge of the bioprinted structures; also, cells with defined polarity could be noted. Bioprinted astrocytes cultivated in NSC medium presented different patterns of marker expression when compared to those cultivated in AST medium. When cultured in NSC medium, SOX2-positive nuclei were only observed in 3D-bioprinted reactive astrocytes at 10 dpp, while this was noted at all timepoints for non-reactive astrocytes. In this group, cells formed a net throughout all constructs, as most DAPI-stained nuclei were SOX2 positive (Figure 7).

**Figure 8:**
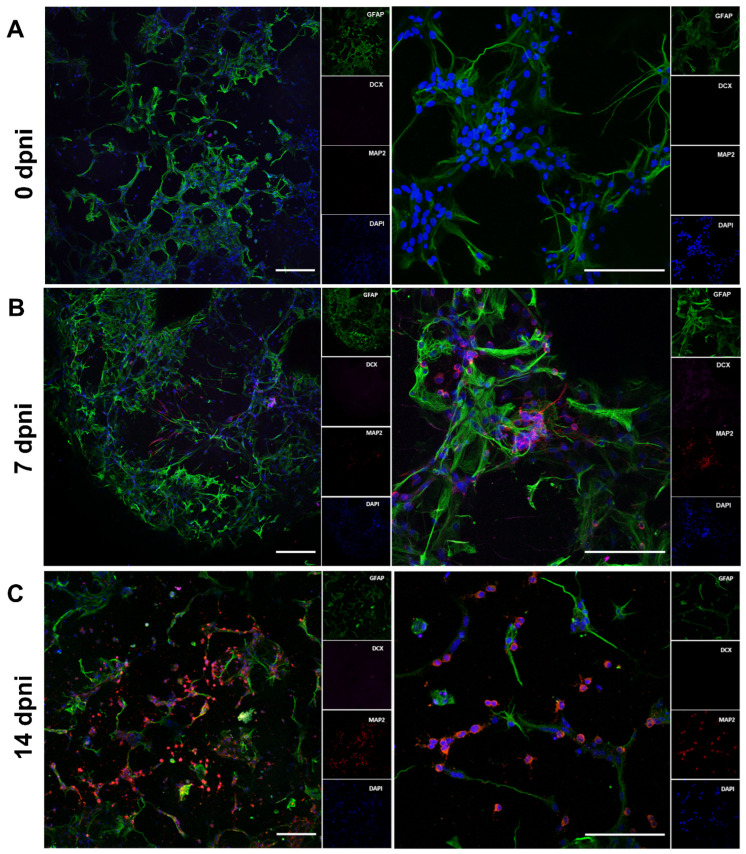
Immunofluorescence analysis of MAP2-positive cells from bioprinted non-reactive astrocytes in neural induction media. No MAP2-positive cells are observed in bioprinted astrocytes before de-differentiated astrocytes’ neuronal differentiation induction (**A**). MAP2-positive cells in bioprinted astrocytes 7 days after de-differentiated astrocytes’ neuronal differentiation induction (**B**). MAP2-positive cells in bioprinted astrocytes 14 days after de-differentiated astrocytes’ neuronal differentiation induction (**C**). Scale bar = 50 µm. dpni = days post neuronal induction.

Based on SOX2 expression, we hypothesized that non-reactive astrocytes cultured in NSC medium underwent a de-differentiation process. To further confirm this, the induction of neuronal differentiation was performed. At 11 dpp, the NSC medium was replaced by a media composition lacking the growth factors EGF and bFGF, with the addition of 10 µM retinoic acid. Before induction, no MAP2-positive cells were observed in the constructs (Figure 8A). Neurogenesis was evaluated by the expression of specific markers of astrocytes and neural stem cells (GFAP), and neurons (MAP2). Seven days after induction, the presence of MAP2-positive cells (Figure 8B) was observed scattered throughout the construct. MAP2-positive cells remained until 14 days after the neuronal differentiation induction started (Figure 8C).

## 4. Discussion

This work presents the development and characterization of bioinks to support in vitro neurogenesis in 3D constructs. Developing bioinks with printability and gelation properties suitable for neural tissue culture is a major challenge in biofabrication [33]. Ideally, bioinks should enable cell proliferation, maintenance of cellular properties and function, and allow stem cell differentiation. For that, some properties are necessary, such as biocompatibility and biodegradation in a controlled way, adequate rheological properties that allow bioprinting and mechanical properties similar to the structure to be designed.

During the bioprinting process, cells plus bioinks are dispensed in a controlled fashion, layer-by-layer, and bioink flow and viscoelastic properties play a crucial role during the fabrication of a construct. High-viscosity bioinks tend to originate higher shear stress during bioprinting, which may reduce cell viability; however, low-viscosity bioinks tend to have more specific bioprinting conditions, such as lower temperature and speed [34].

Low stiffness is a basic characteristic needed for neural cell cultures [33]. Soft constructs allow better oxygen, nutrient, and waste diffusion [33,35]. Furthermore, high stiffness has been reported to limit neurite extension, impair glial cell proliferation, and undesirably direct neural stem cell differentiation [36]. Wu et al. tested 5 to 30% GelMA hydrogels for their ability to support PC12 cells. They found that as the substrate stiffness increased, PC12 cell adhesion decreased. Neurite length first increased in 5 and 10% GelMA, and then decreased in hydrogels containing more than 10% GelMA [37].

The reported stiffness of brain tissue is very soft, ranging from 0.1 to 3 kPa [38]. The mechanical stiffness of reported hydrogels for brain tissue engineering ranges from 1 to 400 kPa [33,36]. The stiffness of the four bioinks presented in this work are 5 to 45 kPa and thus within that range. Both 2.5/2.5G/GMA and 4GMA bioinks also presented low viscosity and good printability, which are essential to increase cell viability.

Softer matrices induce reactivity in astrocytes, which can be reversed by changing matrix stiffness [39]. Additionally, stiffness can also influence stem cell differentiation. Mesenchymal stem cells commit to lineage phenotypes based on sensitivity to matrix elasticity, showing expression and branch localization of the early neuronal marker TUBB3 within 96 h of cultivation [40]. This could explain how astrocytes went through the de-differentiation process so quickly and responded to neuronal differentiation stimuli, showing neuronal markers within seven days of induction.

Bioink porosity is also a major feature to ensure biocompatibility. Shi et al. reported a porous GelMA scaffold in which the inner porous structure was optimized. The inner connected porous structure increased neural stem cell adhesion to the matrix and enhanced neuron differentiation [41]. The SEM pictures confirm that the 2.5/2.5G/GMA and 4GMA bioinks presented higher porosity than the more rigid bioinks (5/5G/GMA and 8GMA), which enhances their biocompatibility.

The bioink composition greatly influences biodegradation, mechanical and cell behavior, including proliferation and differentiation. For example, Zhou et al. used a lipid-bilayer-supported bioprinting model to produce Matrigel^®^ cell-laden constructs, demonstrating early neural developmental interactions [42]. Gelatin and GelMA are widely used in tissue engineering and have already been proven to be compatible with many cells, including neural cells [43,44,45].

Despite the good mechanical properties, we faced biocompatibility problems in all bioinks tested. The human neuroblastoma cell line SH-SY5Y is a very common model in the literature for neurotoxicity studies [46]. When using SH-SY5Y mixed with the bioinks, it was already possible to verify relatively low cell viability in long-term cultures. Mixing Geltrex^TM^ with the bioinks (1:1, *v*/*v*) was enough to increase long-term cell viability. This was more evident when we moved to iPSC-induced hNPCs, and the addition of Geltrex^TM^ to the bioink was essential to maintain cell viability and proliferation for six days. This result follows previous data in the literature [47]. Combining bioinks with extracellular matrix materials such as Matrigel^®^ or Geltrex™ improved neural cell adhesion and proliferation, providing a beneficial environment for cell differentiation phenomena while maintaining the bioinks’ printability features [47].

To verify if the bioinks evaluated here were able to maintain cellular properties, we analyzed gene expression. In SH-SY5Y cells, 2.5/2.5G/GMA_Gx increased the vesicular glutamatergic transporter 1 (vGlut1) expression. Although there is very little data in the field, previous reports suggested that although neuronal maturation is mechanosensitive, neuronal subtype differentiation is not [48]. Since vGlut1 is one of the most specific markers for neurons using glutamate as a neurotransmitter and its activity changes the filling level of synaptic vesicles and modulates the efficiency of excitatory neuro-transmission [49], it is reasonable to think that the 2.5/2.5G/GMA_Gx bioink could be used to drive neurons into a glutamatergic pattern.

Moving to hNPCs, both biocompatible bioinks 2.5/2.5G/GMA_Gx and 4GMA_Gx promoted a reduction in NANOG expression levels. However, since the expression of the neural stem cell (NESTIN) and pluripotency (SOX2) markers was not modified, it seems that hNPC stemness potential was preserved when cultivated in both bioinks. Additionally, the neuronal marker TUBB3 was not upregulated, suggesting that cells remained in an undifferentiated state. It is interesting to mention that light stimulation has been reported to promote neural stem cell neuronal differentiation. Zhu et al. stimulated neural stem cells on 3D-printed scaffolds with a red laser from 15 to 90 s. They found that cell proliferation and the synthesis of reactive oxygen species were increased after one day of culture. After 14 days, neural stem cells increased their neuronal differentiation, while glial differentiation was suppressed [50]. Although we did not find any major changes in hNPCs, the effect of UV exposition during bioink crosslinking remains to be addressed.

The final goal of this work was to use the bioink for bioprinting 3D scaffolds able to support neurogenesis. The bioink 4GMA_Gx was chosen for its ability to fulfil the required criteria: maintain cell viability, minor changes in stemness, and good printability (in comparison with 2.5/2.5G/GMA_Gx, 4GMA_Gx was more easily printed). We speculate if this could be due to lower gelation temperatures and viscosity, which prevent extrusion clogging at room temperature. hNPCs were successfully bioprinted in the 4GMA_Gx composition and cultured for more than 50 days in neural maturation media. During this time, we observed the formation of complex structures with different cell morphologies. At 28 dpp, we identified cells positive for GFAP and NESTIN, which are both known neural progenitor markers, as well as TUBB3. The presence of NESTIN in addition to GFAP- or TUBB3-positive cells, or both at the same time, suggests cells at different stages of maturation, indicating that the hNPCs are directed towards a neuronal, rather than glial, fate [16,51,52,53].

It is well known that astrocytes in the injured CNS become reactive, and a percentage of them undergo a process of de-differentiation, acquiring neural stem cell properties [27]. Although this process is not completely understood, the de-differentiation process can be recapitulated in vitro by a scratch assay. Mature astrocytes become reactive and can acquire a stem cell-like state due to the effect of diffusible factors released from scratch-insulted astrocytes [54]. The 4GMA_Gx bioink supported the de-differentiation process of scratch-induced reactive bioprinted astrocytes cultured in NSC media as verified by the expression of SOX2. Most interestingly, we verified that, in the same conditions, non-reactive bioprinted astrocytes also underwent a de-differentiation process, expressing high levels of GFAP and SOX2 [55]. That was confirmed by inducing neuronal differentiation by adding retinoic acid combined with deprivation of growth factors. SOX2-positive astrocytes were able to generate MAP2-positive cells in seven days. It has been described that the presence of EGF and FGF in the culture medium incites a de-differentiation response, verified by the presence of NESTIN [56,57,58,59]. Non-reactive astrocytes are not reported to generate SOX2-positive cells in vitro when cultivated in a 2D environment with a conventional culture medium, such as AST medium. The observation of astrocytic de-differentiation and the presence of SOX2-positive nuclei in 3D-bioprinted astrocytes, as well as their ability to generate MAP2-positive cells within seven days of differentiation induction, leads us to suggest that the 4GMA_Gx bioink generated a suitable environment to support the de-differentiation response in 3D-bioprinted murine astrocytes.

## 5. Conclusions

Our data indicate that both bioinks, 2.5/2.5G/GMA_Gx and 4GMA_Gx, can support neural cell survival and modulate neural differentiation. The 4GMA_Gx bioink can support neurogenesis from both hNPC and de-differentiated murine astrocytes. The ability of the bioinks to modulate these processes suggests their use in developing synthetic platforms for neural tissue development studies as well as the production of neural tissue replacement and regeneration.

## Figures and Tables

**Table 1:** Bioink compositions.

Bioink	Acronym
2.5/2.5 wt.% Gelatin/GelMA	2.5/2.5G/GMA
5/5 wt.% Gelatin/GelMA	5/5G/GMA
4.0 wt.% GelMA	4GMA
8.0 wt.% GelMA	8GMA
1:1 (*v*/*v*) Geltrex^TM^ + 5/5 wt.% Gelatin/GelMA *	2.5/2.5G/GMA_Gx
1:1 (*v*/*v*) Geltrex^TM^ + 8.0 wt.% GelMA **	4GMA_Gx

* Final Gelatin/GelMA concentration is 2.5/2.5 wt.% Gelatin/GelMA. ** Final GelMA concentration is 4.0 wt.% GelMA

**Table 2:** Primers sequences.

Gene	Sequence	Forward	Reverse
*GAPDH*	NM_001256799.3	GTGGTCTCCTCTGACTTCAAC	CCTGTTGCTGTAGCCAAATTC
*ACTB*	NM_001101.5	TCCACGAAACTACCTTCAACTC	CAGTGATCTCCTTCTGCATCC
*SOX2*	NM_003106.4	TACAGCATGTCCTACTCGCAG	GAGGAAGAGGTAACCACAGGG
*NESTIN*	NM_006617.2	AGAGAGCGTAGAGGCAGTAA	GGTGCTTGAGTTTCTGGAGAT
*NANOG*	NM_001297698.2	GCAAATGTCTTCTGCTGAGATG	CTTTGGGACTGGTGGAAGAA
*TUBB3*	NM_001197181.2	AGTATCCCGACCGCATCAT	AGTAGGTCTCATCCGTGTTCTC
*MAP2*	NM_001039538.2	TGGTGCCGAGTGAGAAGAAG	AGTGGTTGGTTAATAAGCCGAAG
*vGlut1*	NM_020309.4	CGACGACAGCCTTTTGTGGT	GCCGTAGACGTAGAAAACAGAG

*GAPDH*: Glyceraldehyde 3-phosphate dehydrogenase, *ACTB*: Beta-actin, *SOX2*: sex determining region Y-box 2, *NESTIN*: neuroepithelial stem cell protein, *NANOG*: Nanog Homeobox, *TUBB3*: tubulin beta 3 class III, *MAP2*: microtubule associated protein 2, *vGlut1*: Vesicular glutamate transporter 1.

**Table 3:** Compressive modulus of the bioinks analyzed.

Bioink	Compressive Modulus (kPa)
4.0 wt.% GelMA ^1^	5.8 ± 0.9
8.0 wt.% GelMA ^1^	8.5 ± 1.8
2.5/2.5 wt.% Gelatin/GelMA ^1^	8.3 ± 1.6
5/5 wt.% Gelatin/GelMA ^1^	7.5 ± 1.3
4.0 wt.% GelMA ^2^	6.6 ± 0.9
8.0 wt.% GelMA ^2^	25.6 ± 2.3
2.5/2.5 wt.% Gelatin/GelMA ^2^	12.4 ± 2.3
5/5 wt.% Gelatin/GelMA ^2^	45.2 ± 8.6

^1^ compressive modulus calculated at 0.05–0.25% region. ^2^ compressive modulus calculated at 10–20% region.

## Data Availability

Data available on request. The data presented in this study are available on request from the corresponding author.

## Supplementary Materials

The following supporting information can be downloaded at: https://www.mdpi.com/article/10.3390/pharmaceutics15020627/s1, Figure S1: A representation of the materials and apparatus used to manufacture the hydrogels. Example of a sample of Gelatin/GelMA (2.5% and 2.5% m/m) after the UV crosslinking process (A), a sample stamped for mechanical tests (B), and a sample inserted in the equipment for determination of mechanical properties in compression regime (C); Figure S2: A summary and flow of all performed biological tests. GF: growth factors, RA: retinoic acid; Figure S3: hNPCs successfully differentiated from hiPSCs. Immunofluorescence for OCT4 (octamer-binding transcription factor 4) and NESTIN (neuroepithelial stem cell protein) markers in hNPCs on passage 4. Very faint presence of OCT4, known pluripotency marker, in immunofluorescence (A). Strongly marked cells for NESTIN (B). Gene expression analysis of pluripotency and neural progenitor markers in generated hNPC cell line relative to hiPSC (C). Scale bar = 50 µm. *OCT4*: octamer-binding transcription factor 4; *NANOG:* Nanog Homeobox; *SOX2*: sex determining region Y-box 2; *NESTIN*: neuroepithelial stem cell protein; *PAX6*: paired box 6; *FOXG1*: Forkhead Box G1; *TBR2*: T-box brain protein 2; Figure S4: hNPC morphology in bioink. In Geltrex^TM^ added bioink and in the 2D it is possible to see cell extensions (arrows) in comparison to the round shape observed in other bioink without Geltrex^TM^, here represented by 5/5G/GMA. Scale bar = 100 µm; Figure S5: Bioprinted hNPC morphology Light microscope image of 3D bioprinted hNPC in neural maturation media 1 dpp. It is noticeable that some cells present a more elongated morphology (A). Light microscope images in of 3D bioprinted hNPC in neural maturation media 10 dpp. Cell clusters have increased in size and connected through cell elongations (B). Light microscope images in of 3D bioprinted hNPC in neural maturation media 54 dpp. Cell clusters and neuron cells throughout the construct (C). Photo taken by a cellphone of bioprinted constructs 61 ddp in 24 well plate. The bigger cell clusters are noticeable as white dots and connected lines (D). Scale bar = 100 µm. dpp = days postprinting; Figure S6: Immunofluorescence analysis of GFAP, SOX2, DCX positive cells in bioprinted murine astrocytes under influence of AST culture media. When cultivated with AST media, non-reactive bioprinted astrocytes presented a round morphology in contrast with the reactive astrocytes star shape. SOX2 expression was present in reactive astrocytes at day 5 post printing, which was not observed in the subsequent timepoint or any other experimental groups. No groups showed expression of doublecortin (DCX). Scale bar (bright-field) = 100 µm. Scale bar (fluorescence) = 50 µm. dpp = days post-printing.
