# Supplementary material for: A Gelatin Methacrylate-Based Hydrogel as a Potential Bioink for 3D Bioprinting and Neuronal Differentiation
Source: Pharmaceutics. 2023 Feb 13;15(2):627. doi: 10.3390/pharmaceutics15020627 (PMC9959598; doi:10.3390/pharmaceutics15020627)
Supplement: Supplementary file 1 [file pharmaceutics-15-00627-s001.zip › pharmaceutics-2150044-supplementary.pdf]

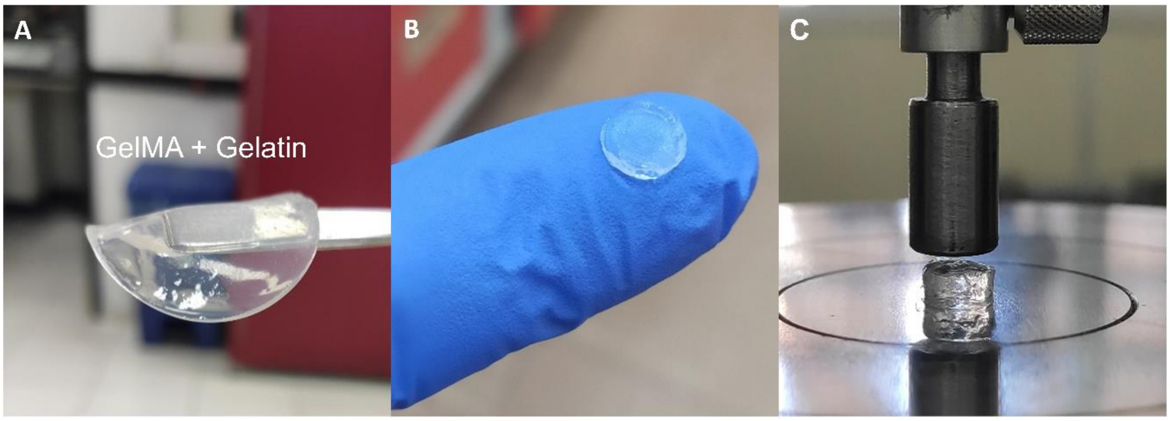

Figure S1. A representation of the materials and apparatus used to manufacture the hydrogels. Example of a sample of Gelatin/GelMA (2.5% and 2.5% m/m) after the UV crosslinking process (A), a sample stamped for mechanical tests (B), and a sample inserted in the equipment for determination of mechanical properties in compression regime (C).

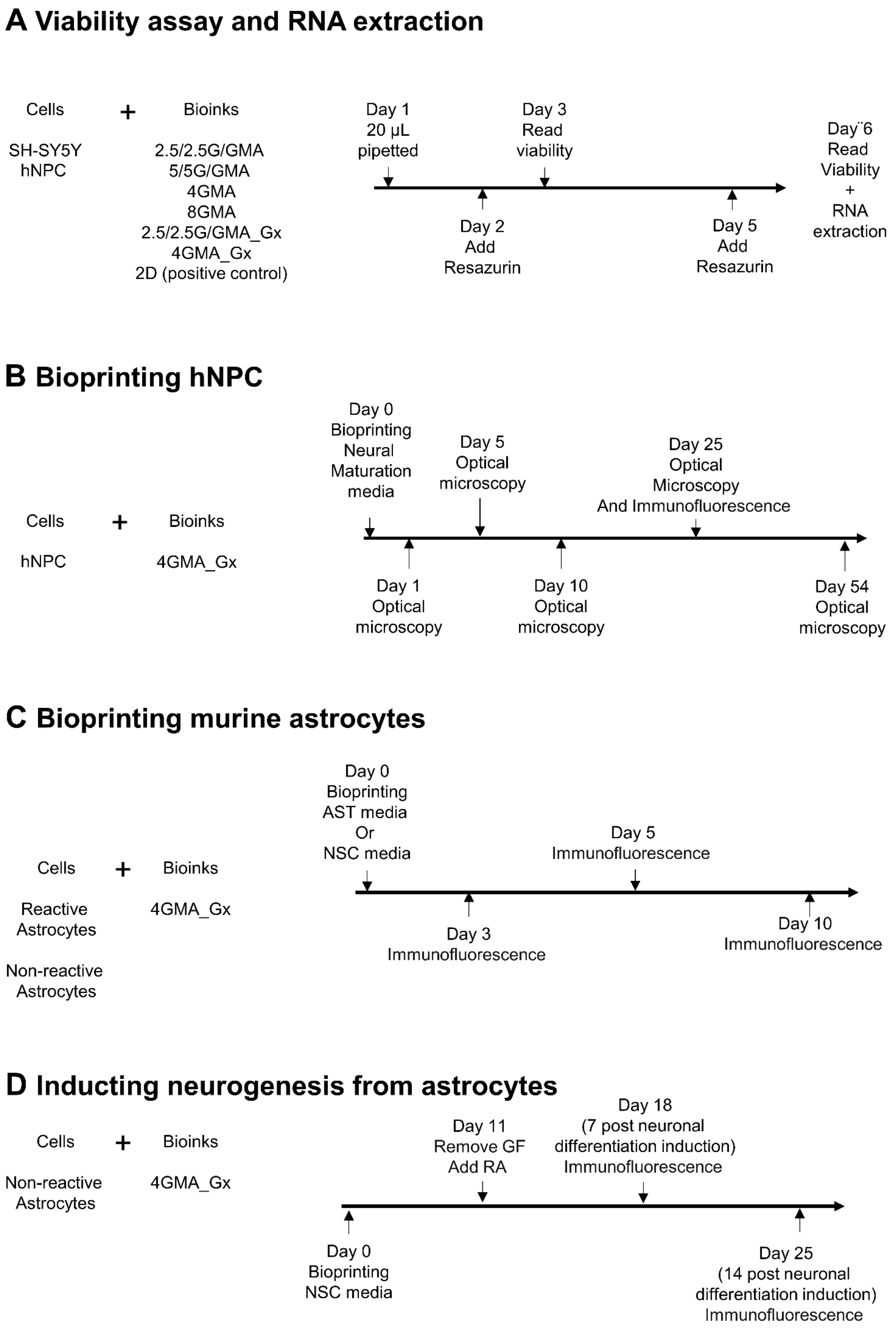

Figure S2. A summary and flow of all performed biological tests. GF: growth factors, RA: retinoic acid.

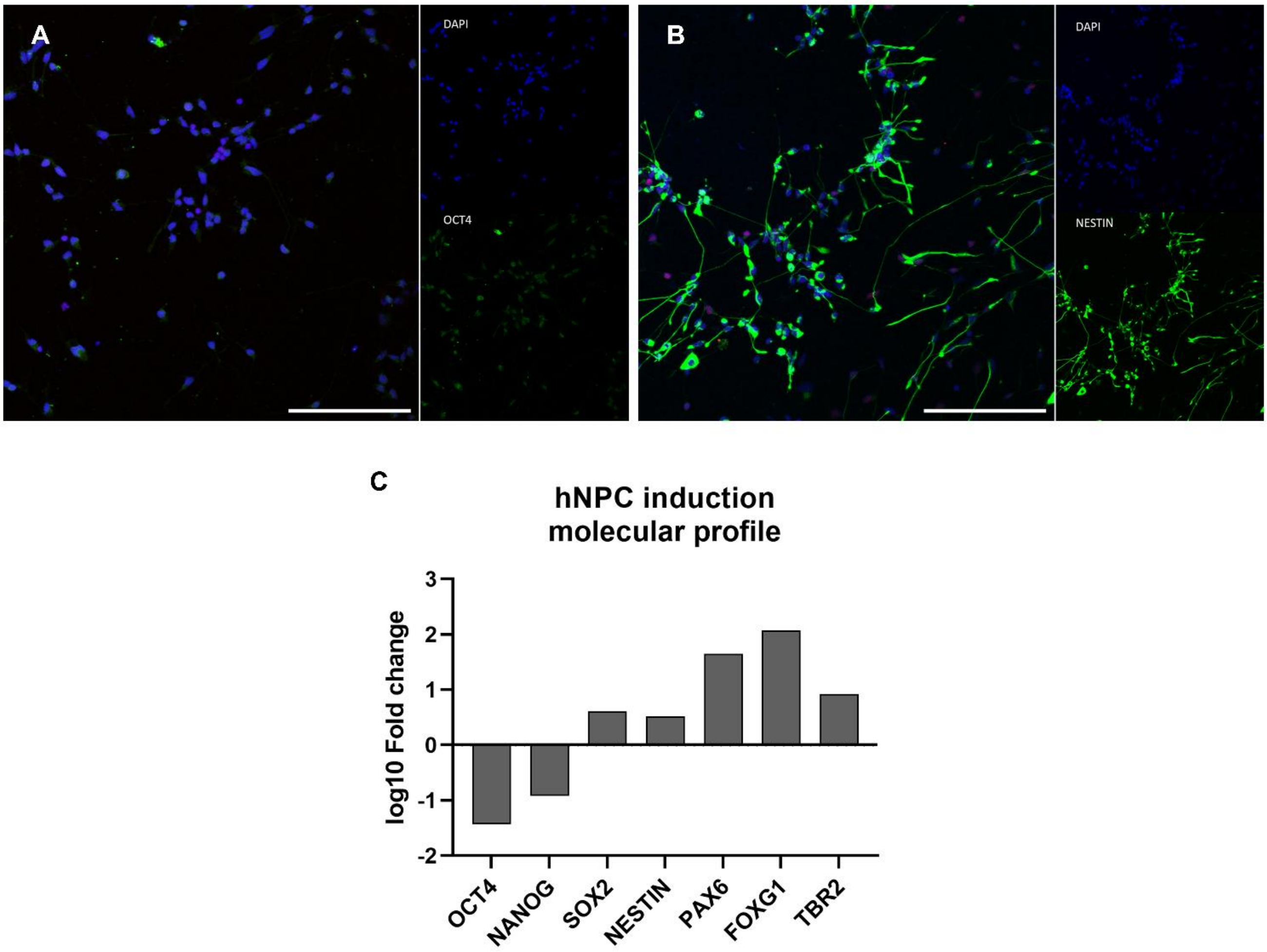

Figure S3. hNPCs successfully differentiated from hiPSCs. Immunofluorescence for OCT4 (octamer-binding transcription factor 4) and NESTIN (neuroepithelial stem cell protein) markers in hNPCs on passage 4. Very faint presence of OCT4, known pluripotency marker, in immunofluorescence (A). Strongly marked cells for NESTIN (B). Gene expression analysis of pluripotency and neural progenitor markers in generated hNPC cell line relative to hiPSC (C). Scale bar = 50 μm. *OCT4*: octamer-binding transcription factor 4; *NANOG:* Nanog Homeobox; *SOX2*: sex determining region Y-box 2; *NESTIN*: neuroepithelial stem cell protein; *PAX6*: paired box 6 ; *FOXG1*: Forkhead Box G1; *TBR2*: T-box brain protein 2.

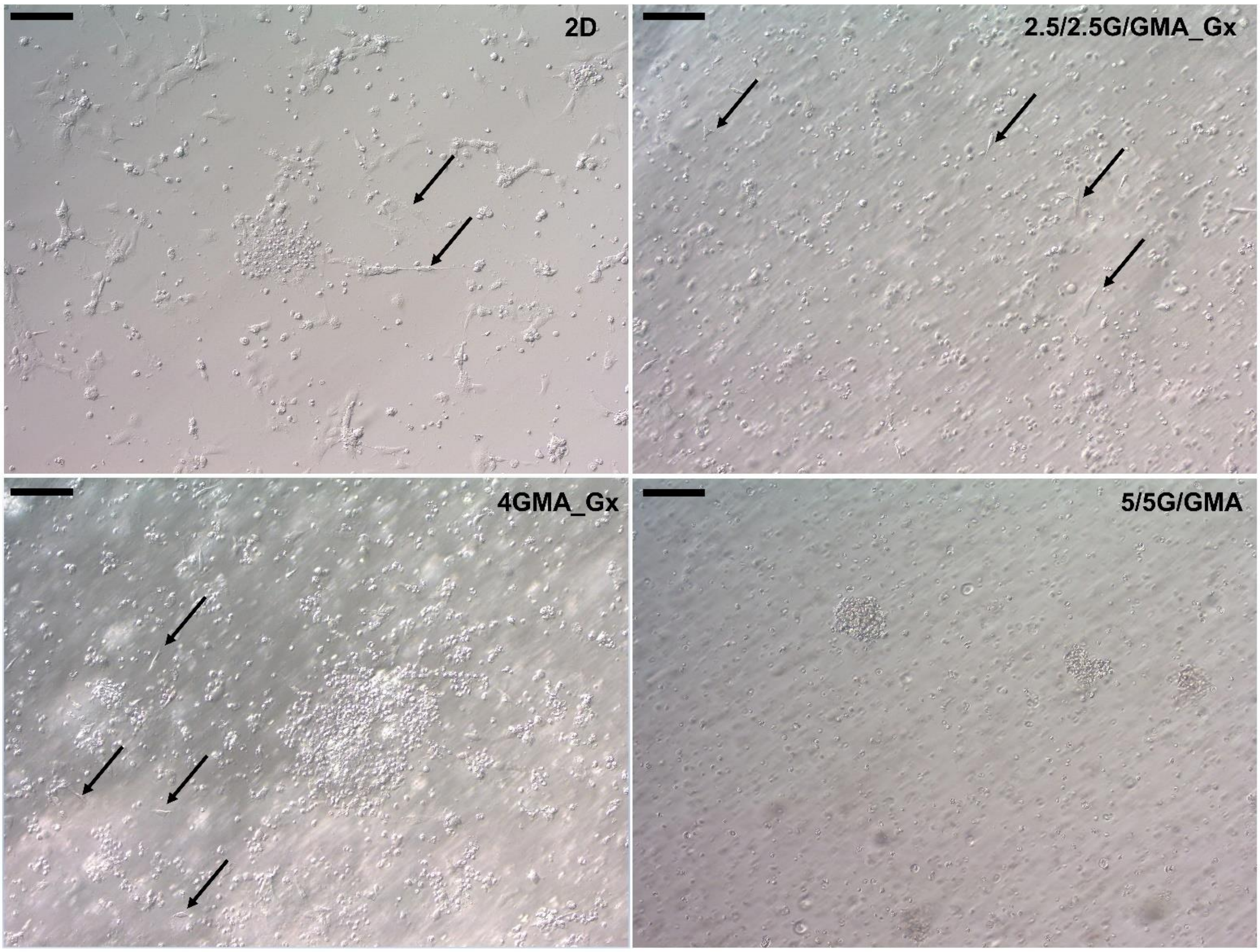

Figure S4. hNPC morphology in bioink. In GeltrexTM added bioink and in the 2D it is possible to see cell extensions (arrows) in comparison to the round shape observed in other bioink without Geltrex™, here represented by 5/5G/GMA. Scale bar = 100 μm.

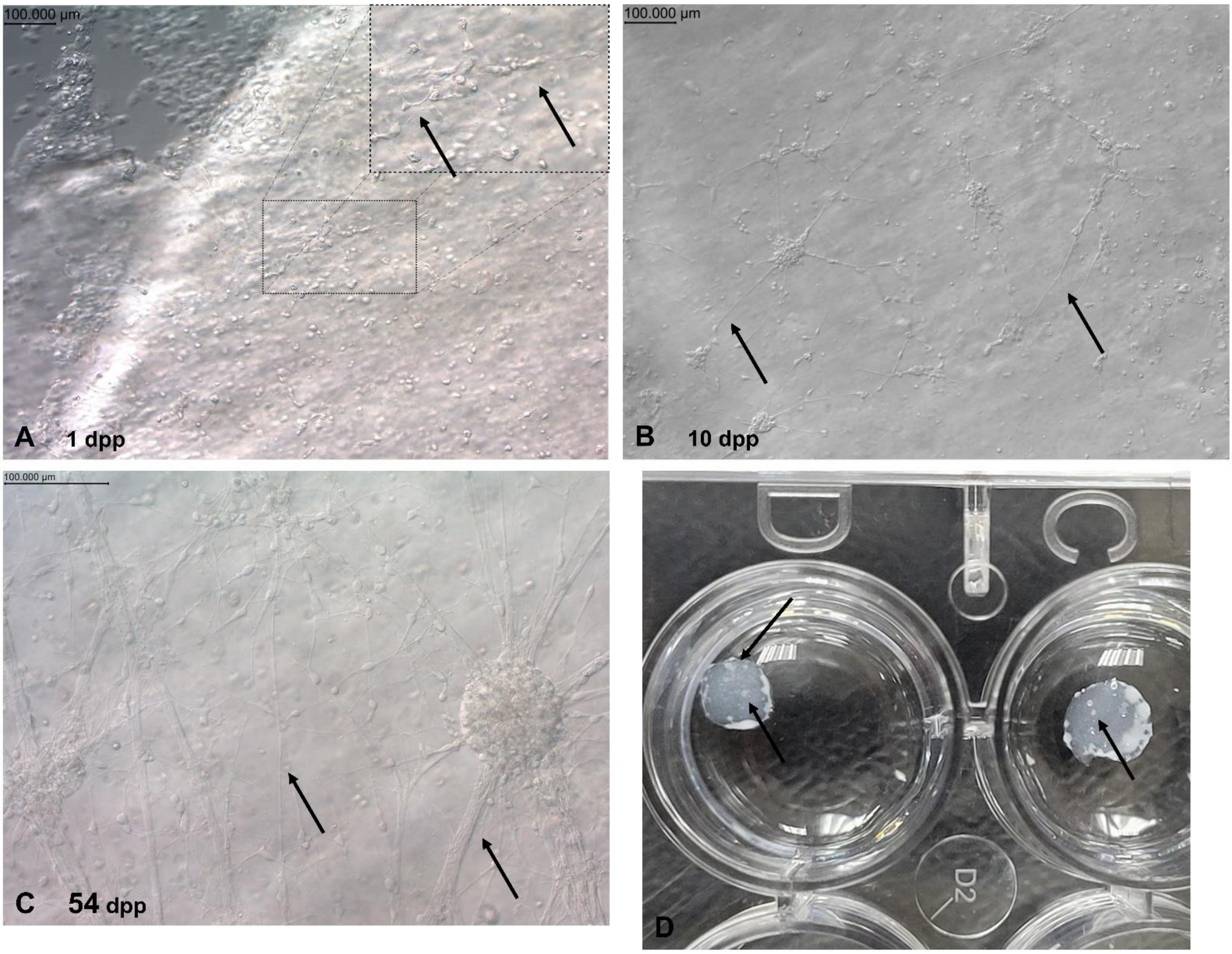

Figure S5. Bioprinted hNPC morphology Light microscope image of 3D bioprinted hNPC in neural maturation media 1 dpp. It is noticeable that some cells present a more elongated morphology (A). Light microscope images in of 3D bioprinted hNPC in neural maturation media 10 dpp. Cell clusters have increased in size and connected through cell elongations (B). Light microscope images in of 3D bioprinted hNPC in neural maturation media 54dpp. Cell clusters and neuron cells throughout the construct (C). Photo taken by a cellphone of bioprinted constructs 61 ddp in 24 well plate. The bigger cell clusters are noticeable as white dots and connected lines (D). Scale bar = 100µm. dpp = days postprinting.

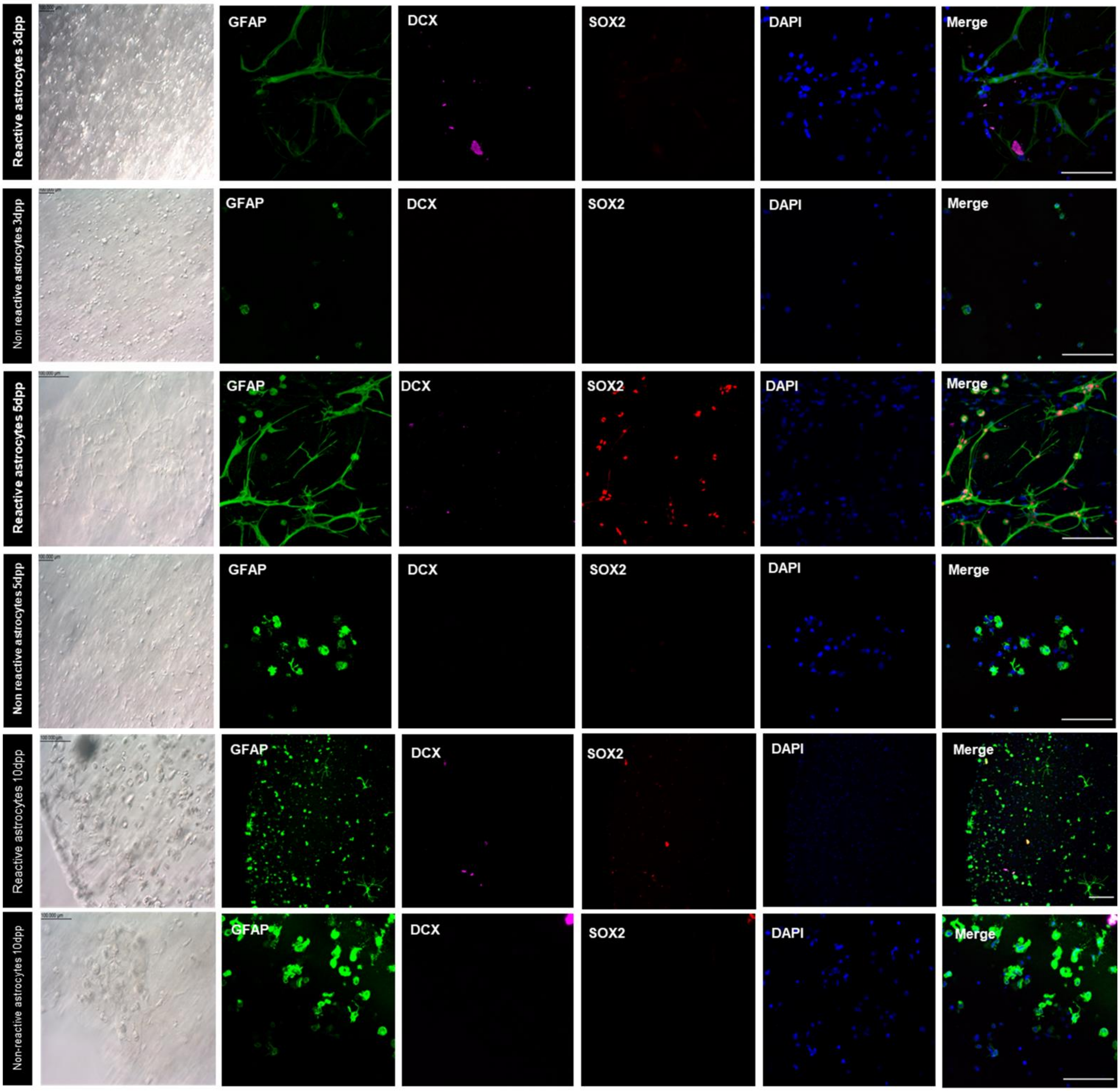

Figure S6. Immunofluorescence analysis of GFAP, SOX2, DCX positive cells in bioprinted murine astrocytes under influence of AST culture media. When cultivated with AST media, non-reactive bioprinted astrocytes presented a round morphology in contrast with the reactive astrocytes star shape. SOX2 expression was present in reactive astrocytes at day 5 post printing, which was not observed in the subsequent timepoint or any other experimental groups. No groups showed expression of doublecortin (DCX). Scale bar (bright-field) = 100µm. Scale bar (fluorescence) = 50µm. dpp = days post-printing.
